# Stepping stones in DNA sequencing

**DOI:** 10.1002/biot.201200153

**Published:** 2012-08-08

**Authors:** Henrik Stranneheim, Joakim Lundeberg

**Affiliations:** 1 Science for Life Laboratory, KTH Royal Institute of TechnologyStockholm, Sweden; 2 Department of Molecular Medicine and Surgery, Karolinska InstitutetStockholm, Sweden

**Keywords:** DNA, Genomics, Nanopore sequencing, Parallel sequencing, Sequencing

## Abstract

In recent years there have been tremendous advances in our ability to rapidly and cost-effectively sequence DNA. This has revolutionized the fields of genetics and biology, leading to a deeper understanding of the molecular events in life processes. The rapid technological advances have enormously expanded sequencing opportunities and applications, but also imposed strains and challenges on steps prior to sequencing and in the downstream process of handling and analysis of these massive amounts of sequence data. Traditionally, sequencing has been limited to small DNA fragments of approximately one thousand bases (derived from the organism's genome) due to issues in maintaining a high sequence quality and accuracy for longer read lengths. Although many technological breakthroughs have been made, currently the commercially available massively parallel sequencing methods have not been able to resolve this issue. However, recent announcements in nanopore sequencing hold the promise of removing this read-length limitation, enabling sequencing of larger intact DNA fragments. The ability to sequence longer intact DNA with high accuracy is a major stepping stone towards greatly simplifying the downstream analysis and increasing the power of sequencing compared to today. This review covers some of the technical advances in sequencing that have opened up new frontiers in genomics.

## 1 Introduction

Fully understanding the language of DNA requires the complete determination of the order of bases in the genome of humans (or other organisms of interest). Acquiring that knowledge promises to yield more complete insights into biological variations and etiology of diseases. In the dawn of sequencing, reading the order of the four bases of DNA was a cumbersome process. Sequencing was made possible, although still difficult, by the introduction of the chemical degradation method of Maxam and Gilbert [1] and Sanger's chain-termination sequencing method [2] at the end of the 1970s. The latter proved to be more useful and was to be the dominant DNA sequencing technique for almost three decades, propelled by the Human Genome Project (HGP), and is still considered by many the “gold standard”. The commercial launch of massively parallel DNA sequencing instruments in 2005 initiated a paradigm shift powered by new DNA sequencing techniques that inspired researchers to address bolder questions in genome-wide experiments. Recent years have seen many new contenders in the field of massively parallel sequencing. Notably, innovative novel sequencing methods using single molecules of DNA and real-time detection have emerged. Currently, these novel approaches are complementing existing sequencing platforms, but they have a long way to go before replacing massively parallel sequencing.

**Figure 1:**
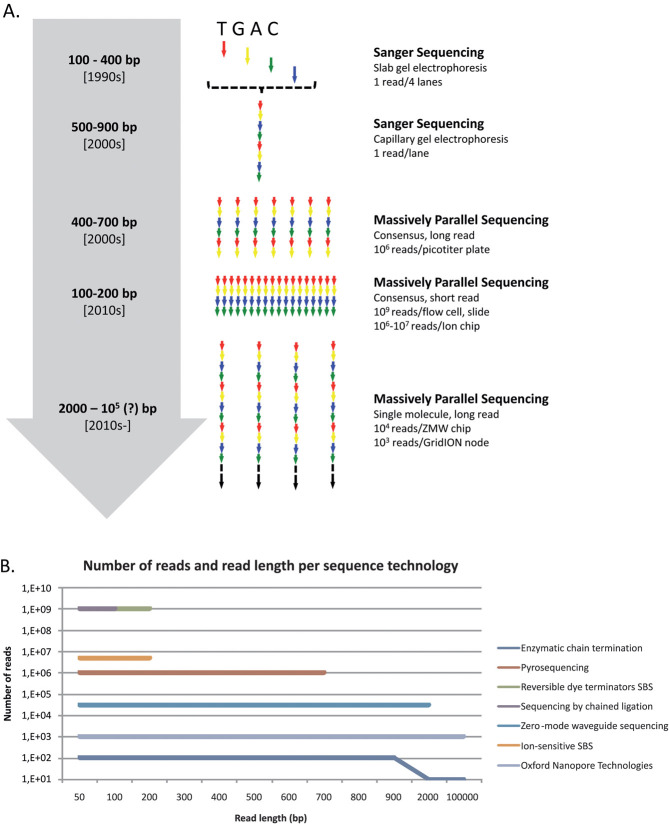
(**A**) Changes of read length and degree of parallelism in sequencing technologies since the 1990s up to the present. (**B**) Number of reads and read length per sequencing technology.

The most commonly used DNA sequencing technologies, and their challenges and limitations, are described below. A summary table of the features of each technology is provided in Table 1 while the number of sequence reads and read lengths are shown in Fig. 1.

**Table 1:** Overview of sequencing techniques and their specificationa)

		Sequencing principle	Sample preparation	Detection principle	Major error modality	Read length (bp)	Sample throughput
First generation sequencing	Sanger sequencing	Enzymatic chain termination	Cloning/PCR	Optical	Substitutions	900	Low

Consensus sequencing	454	Pyrosequencing	Emulsion PCR	Optical	Indels	700	Medium
	Illumina/Solexa	Reversible dye terminators SBS	Bridge amplification	Optical	Substitutions	100	High
	SOLiD	Sequencing by ligation	Emulsion PCR	Optical	Substitutions	75	High
	Complete Genomics	Sequencing by ligation	Restriction/circularization/RCA	Optical	NA	35	High
	Ion Torrent	Ion-sensitive SBS	Emulsion PCR	Electronic	Indels	200	Medium

Single-molecule sequencing	Helicos	Reversible single-dye terminators SBS	No amplification	Optical	Deletions	35	Medium
	Pacific Biosciences	ZMW sequencing	No amplification	Optical	Indels	>2000	Medium
	Nanopore sequencing	Ionic current shift	NA	Electronic/Optical	NA	NA	NA

a) NA, not available; RCA, rolling circle amplification.

## 2 Sanger sequencing – chain-termination sequencing

The ingenuity of the chain-termination technique lies in the use of chain-terminating nucleotides, dideoxynucleotides that lack a 3'-hydroxyl group, restricting further extension of the copied DNA chain. Early Sanger sequencing required the sequencing process to be split into four separate reactions. Each reaction involves a single-stranded DNA template, a DNA primer and a DNA polymerase in the presence of a mixture of the four unmodified nucleotides, one of which is labeled, and a type of modified chain-terminating nucleotide. Fragments of varying length are synthesized after primer hybridization and polymerase extension, all having the same 5' end but terminated by a chain-terminating nucleotide at the 3' end. Adding only a fraction of the terminating nucleotide ensures the random incorporation of dideoxynucleotides in only a small subset of molecules. Since different chain-terminating nucleotides are used in the four sequencing reactions, all combinations of termination can be produced. The generated 3'-terminated DNA templates are then heat-denatured and fractionated by gel-electrophoresis, running products of all four sequencing reactions in parallel [2]. Using radioactive or, more recently, fluorescent labeling to visualize the bands enables the sequence of the original DNA template to be determined by following the migration order of successively larger fragments in the gel.

Several enhancements have been made to the original method developed by Sanger. These include: labeling the chain-terminating nucleotides with spectrally distinct fluorescent dyes enabling: a single tube and lane to be used in the fragment generation and fractionation steps, respectively [3, 4]; elimination of the need to cast gels by using capillary gel electrophoresis [5, 6]; and automation of the protocol, leading to increases in parallelization, reproducibility and throughputs [7]. Sanger sequencing is still widely used today for many applications, particularly validation of genetic variants and in cases where high quality reads of 300–900 bases are needed. However, the major advances in sequencing technology in recent years have not been related to the mature Sanger sequencing method, but to the rapidly evolving massively parallel sequencing methods.

## 3 Massively parallel sequencing – consensus sequencing

The most widely used sequencing platforms in genetic research today are the massively parallel sequencing platforms, which have inherited many features from Sanger sequencing, such as the use of polymerases for synthesis, modified nucleotides and fluorescent detection. Another feature is that they require the DNA to be clonally amplified forming a consensus template prior to sequencing. These sequencing methods have been called next generation sequencing, high-throughput sequencing methods, or second-generation sequencing. However, as the sequencing technologies continue to develop, it is hard to keep referring to old and novel emerging sequencing technologies as belonging to a certain generation. Therefore, it is preferable to categorize them by their most prominent common feature, such as being massively parallel or single-molecule sequencing methods. Currently, there are five competing massively parallel sequencing technologies, each with specific strengths and weaknesses. These are described and discussed below.

### 3.1 Pyrosequencing

Melamede originally outlined the concept of sequencing-by-synthesis (SBS) in 1985, in a report of efforts to detect nucleotide incorporation events by measuring nucleotide absorbance [8]. Unaware of Melamede's findings, Nyrén conceived another SBS approach using bioluminescence instead of absorbance in 1986 [9], which led (after another 12 years of experiments) to the introduction of pyrosequencing [10]. Pyrosequencing technology was able to complement Sanger sequencing and was further developed by 454 Life Sciences, founded by Jonathan Rothberg. In 2005, Rothberg and colleagues [11] released the first proof-of-concept paper demonstrating a massively parallel pyrosequencing approach, yielding a tremendous increase in sequence capacity, and thereby transforming the way sequencing was conducted.

In SBS, the event that is detected is the incorporation of nucleotides into growing DNA strands. As the nucleotides are incorporated by the polymerase, pyrophosphate (PPi) and protons are generated. In pyrosequencing, the PPi is used in an enzymatic cascade to generate a light burst. The PPi is converted by ATP sulfurylase to ATP, which is then used by the firefly enzyme luciferase to generate photons, providing a light signal that is proportional to the number of nucleotides being incorporated. The challenge is to detect the flashes of light from each unique DNA template while sequencing numerous templates in parallel. Spatially separating each sequencing reaction on beads deposited in small wells on a picotiter plate elegantly solved this problem.

Massively parallel pyrosequencing begins with fragmentation of the DNA and adaptor ligation. Single-stranded DNA templates are then bound on beads and emulsion PCR is performed, clonally amplifying each DNA template in aqueous microreactors isolated by oil. The emulsion is then broken and the beads carrying DNA are separated from empty beads in a process called enrichment. The enriched beads are deposited in the small wells on the picotiter plate together with primer and DNA polymerase. Ideally, only one of these beads will fit into each well. Smaller beads are also added, carrying the enzymes responsible for the light generation using PPi. Nucleotides are then passed over the substrate in a laminar flow of solutions applied in a predetermined order, and the flashes of light in each well representing incorporation events are recorded. Efficient removal of reaction by-products, which could otherwise perturb the sequencing reaction, is facilitated by the laminar flow. Hence, the sequence of the DNA templates is determined from the knowledge of their location, the order of the flow of nucleotides and records of each flash of light from each well [10–12]. The major drawback using this approach are difficulties in sequencing stretches of identical nucleotides (homopolymeric regions) longer than approximately five nucleotides due to the nonlinear light response they generate [10].

The comparable read lengths and increased sequence capacity compared to traditional Sanger sequencing made the massively parallel pyrosequencing ideal for de novo sequencing, re-sequencing of genomes and metagenomic studies [12]. James Watson, who contributed to solving the structure of DNA, had his genome sequenced by massively parallel pyrosequencing in 2008 [13]. The major impact of this sequencing platform can be realized by comparing the sequencing of Watson's genome – completed in only two months at a cost of US$1 million [13] – to the HGP endeavor, which took 11 years and cost US$3 billion [14]. Another milestone using massively parallel pyrosequencing is the work carried out on the Neanderthal genome by Pääbo and co-workers [15]. Recently, the read length of the platform has increased from 450 bases to rival that of routine Sanger sequencing at 700 bases. Massively parallel pyrosequencing transcended Sanger sequencing by enormously increasing throughput. However, the research community seemed to have developed an insatiable appetite for sequence data, which is not easily satisfied using bioluminescent SBS.

### 3.2 Reversible dye terminator SBS

A fundamental gain in sequencing throughput was achieved when the second massively parallel sequencing system to be commercialized was launched in 2006. The technology is based on SBS using four colored reversible dye termination nucleotides and was first outlined in 2001 by a small company, Solexa [16, 17], which was later acquired by Illumina [18].

SBS using reversible dye termination begins with fragmentation of the DNA and ligation of adaptors. Instead of using emulsion PCR to spatially separate each DNA template, as in the massively parallel pyrosequencing approach, another technique is employed. The DNA templates are hybridized via the adaptors to a planar surface, where each DNA template is clonally amplified by solid-phase PCR, also known as bridge amplification. This creates a surface with a high density of spatially distinct clusters, each cluster of which contains a unique DNA template. These are primed and sequenced by passing the four spectrally distinct reversible dye terminators in a flow of solution over the surface in the presence of a DNA polymerase. Only single base extensions are possible due to the 3' modification of the chain-termination nucleotides, and each cluster incorporates only one type of nucleotide, as dictated by the DNA template forming the cluster. The incorporated base in all clusters is detected by fluorescence imaging of the surface before chemical removal of the dye and terminator, generating an extendable base that is ready for a new round of sequencing. The most common sequencing errors produced in reversible dye termination SBS are substitutions [18, 19].

One of the major drawbacks of reversible dye termination chemistry is the limitation in read length, as it is difficult to reach 100% efficiency of base incorporation and cleavage in each cycle. When the system was launched, it featured a high- quality read length of only 30–36 bases [18, 20], dramatically less than the read lengths offered by both Sanger sequencing and massively parallel pyrosequencing. In fact, it was even lower than that of the “plus-minus” method developed in 1975 by Sanger and Coulson [21], but a dramatic increase in sequence data output compensated for this drawback. Substantial improvements in both read length and throughput have been achieved since its introduction, enabling reversible dye termination SBS to become the most successful massively parallel sequencing platforms. The short read length initially prohibited its use in large de novo assembly applications, but made it suitable for re-sequencing and data counting applications, e.g. sequencing of transcriptomes, RNA-Seq and sequencing of DNA fragments involved in protein-DNA interactions, ChIP-Seq [18]. Improvements in read length and development of novel de novo assembly algorithms, such as SOAPdenovo [22], has now enabled the use of reversible dye termination SBS in de novo assembly of large mammalian genomes, e.g. the giant panda genome [23]. Recently, Illumina released its rapid Hiseq 2500 system capable of sequencing a human genome in 27 h, and enabling read lengths up to 150 bases through shorter sequence cycling times.

### 3.3 Sequencing by chained ligation

In nature, DNA ligases are essential enzymes for repairing nicks and breaks in DNA and are involved in high fidelity reactions in both DNA replication and repair [24]. In 2005, Church and Shendure demonstrated that the reaction they catalyze could be used to sequence short DNA fragments immobilized on beads trapped in a polyacrylamide gel in a process called polony sequencing [25]. SOLiD (Sequencing by Oligonucleotide Ligation and Detection), the third commercial massively parallel sequencing platform, based on a more mature version of this technique, reached the market in 2007 [26].

Initially, the SOLiD platform yielded many more bases than the Illumina platform, but the recent upgrades to the Hiseq2000 chemistry make this instrument's yield significantly higher than that for SOLiD. However, in the library preparation steps the SOLiD platform resembles massively parallel pyrosequencing. Clonal amplification is achieved by emulsion PCR after creating an adaptor-ligated fragment library. The enriched beads are then randomly deposited on a glass slide and attached to its surface by a 3' modification introduced on the targets covering the beads. In subsequent sequencing by chained ligation, oligomers of eight nucleotides, octamers, are used as detection probes instead of single nucleotides. Each octamer contains two known bases at the 3' end followed by three degenerate nucleotides and three nucleotides that can hybridize to any other nucleotide, called universal nucleotides. Four spectrally distinct dyes are employed, each of which is carried by four octamers, creating a total probe set of 16 octamers, covering all possible combinations of the two known bases. The dye is attached to the 5' end of each octamer. To initiate the first sequencing cycle, the DNA-covered beads are incubated with a sequencing primer, the 16 octamer probes and ligase. Only a perfectly hybridized probe will be joined to the sequencing primer by the ligase. The probes are imaged to decode the first two bases, and then chemically cleaved to remove the last three 5' bases. This cycle of probe hybridization and ligation, fluorescent imaging and chemical cleavage is repeated ten times, interrogating every fifth base. The created fragment is then stripped from the beads and a second ligation round is performed using a sequencing primer annealing one base upstream of the previous one. Hence, each base is associated with two color calls. Progressively shifting the position of the sequencing primer and repeating the ligation round using ten cycles each time yields color calls, which can be decoded to obtain a linear sequence in color space. Translating from color space to an ordinary nucleotide sequence is most accurately performed by aligning the color space reads to a color space reference genome. As a consequence of the color space alignment, errors can be efficiently corrected since only certain color combinations are allowed, facilitating distinction between nucleotide substitutions, represented by a two-color change in adjacent nucleotides, and sequence errors [26–28]. The most common sequencing errors in sequencing by chained ligation are substitutions [18].

Life Technologies has recently announced a replacement of the cumbersome emulsion library preparation step with a technique called wildfire, which resembles the bridge amplification performed in the Illumina platform. This promises a reduction of the library preparation time as well as a higher density on the chip surface, enabling a higher throughput. The SOLiD system is best suited for re-sequencing projects demanding low error rates [26], transcriptome sequencing and tag counting, e.g. ChIP-Seq, due to its short read length, error correction system and massive output of data [18]. The color space approach efficiently detects sequence errors, but the downstream data analysis has proven to be cumbersome, leading to the development of few pertinent open source tools [29]. However, it is possible to exploit the ligation reaction in sequencing without applying the color space approach.

### 3.4 Sequencing by unchained ligation

In 2010, Complete Genomics Inc published a proof-of-principle paper demonstrating its complete human genome sequencing capabilities, using a sequencing method sharing several features with polony sequencing [25, 30]. Rather than selling sequencing instruments, which has been the dominant business model so far, the company provides an outsourcing service for human DNA sequencing including library preparation, sequencing and basic analysis.

Complete Genomics performs sequencing by unchained ligation using its combinatorial probe-anchor ligation (cPAL) technology. The DNA to be sequenced is fragmented to 500 bases and four adaptors are inserted into each genomic fragment by repeated cutting with restriction enzymes and intramolecular ligation. The resulting circles are then amplified in solution into coils of single-stranded DNA [31], called DNA nanoballs, composed of repeated copies of the original circles. The DNA nanoballs are then randomly attached to ordered spots on a planar surface. Each spot is small enough to contain only one DNA nanoball. Complete Genomics' approach of sequencing by unchained ligation uses anchor probes to find adaptor sites and detection probes consisting of nine bases (nonamers) and four dyes. The anchor probe and detection probe are joined by hybridization and ligation to decode one base each cycle. The nanoballs can be decoded, one nucleotide at a time, on both sides of each adaptor site. Briefly, an anchor oligonucleotide is hybridized to one of the four adaptors, and four probes, each carrying a spectrally distinct dye, are used to interrogate a base position adjacent to the adapter. The dye is connected to a known base at a specific position in the nonamer probe, while the rest of the positions are filled by degenerate nucleotides. Varying the position of the known base in the probe allows positions 1–5 in the DNA template adjacent to the adaptor to be read. Creation of an extended anchor probe by ligation of two anchor probes allows decoding of positions 6–10 adjacent to the adaptor. The anchor-probe complex is stripped off after fluorescent detection of each hybridization and ligation reaction. This resets the system. Hence, each base call is unchained, thereby improving sequence quality because every decoded base is independent of the completeness of earlier cycles. The whole procedure is then repeated for both sides of each adaptor site, and the resulting 10-mers are merged into 35-base-paired-end reads [30].

The reasons that Complete Genomics are keeping their sequencing platform in-house are, at least partly, related to the considerable challenge of mapping and assembling the highly repetitive human genome using short, 35-base-paired-end reads and the rather demanding cPAL library preparation. Illumina has responded to Complete Genomics' initiative by also offering complete human genome sequencing as a service, using reversible dye termination chemistry. It remains to be seen if most sequencing will be performed in-house at hospitals and sequencing centers, or outsourced to sequencing companies.

### 3.5 Ion-sensitive SBS

All massively parallel sequencing platforms described so far use optic detection to decipher a DNA sequence. Exchanging optic detection for electric detection would potentially have several advantages since it would avoid the need for modified nucleotides, expensive optics and the time-consuming acquisition and processing of large amounts of fluorescent image data. Hence, it promises cheaper, space-efficient and more rapid sequencing. Pourmand and colleagues [32] outlined an electric detection SBS approach in 2006, demonstrating that DNA synthesis events could be detected by electric charge perturbations using a voltage-clamp amplifier. In 2010, Rothberg and colleagues [33] demonstrated a novel sequencing platform, based on the work by Pourmand et al. [34], the Ion Torrent, which relies on electric rather than fluorescent signals to obtain sequence data.

The process is very similar to that of the massively parallel pyrosequencing, developed earlier by Rothberg and co-workers. It involves use of the same library preparation steps, with an adapter-ligated fragment library, which is clonally amplified on beads using emulsion PCR. A DNA polymerase is used to incorporate nucleotides on a primed DNA template with a predetermined flow of nucleotides. However, the focus is shifted from the PPi to the other by-product of nucleotide incorporation, the hydrogen ion. The enriched beads from the emulsion PCR are deposited in small wells (each equipped with a sensor capable of detecting free protons) on an ion chip. As the DNA polymerase adds nucleotides to the DNA template hydrogen ions are released, and the resulting free proton shift is detected by the sensor and transformed to an electric signal. The ion chip is based on the most widely used technology to construct integrated circuits, facilitating low-cost, large-scale production and should confer excellent scalability. However, as in massively parallel pyrosequencing, homopolymeric regions give rise to insertions and deletions, indels, due to the nonlinear electric response upon incorporation of more than five nucleotides [33].

Sequencing of Gordon Moore's genome [33] demonstrated that it is possible, but perhaps not recommendable, to use the Ion Torrent system for large mammalian re-sequencing. The Ion Torrent system has a fast sequencing run time of just 2 h, but resembles massively parallel pyrosequencing when it was launched in terms of throughput and read length. It seems most suited for de novo sequencing and re-sequencing of bacterial genomes or sequencing targeted regions of more complex organisms' genomes. The recent outbreak of the Shiga toxin-producing substrain of *Escherichia coli* provided an excellent example of the value of a sequence platform with a modest output but fast sequencing run time when monitoring outbreaks of pathogens [35, 36]. The successor to Ion Torrent is called the Ion Proton, scheduled to be released in 2012, and is based on the same chemistry as its predecessor but is expected to have about 60-fold more wells on its Ion Proton II chip. This massive increase in throughput should enable the sequencing of a human genome within a few hours of sequencing.

### 3.6 Current issues in massively parallel sequencing

The massively parallel sequence methods are quite diverse in terms of sequencing biochemistry, but they share many common features. Their library preparation steps begin with random fragmentation of DNA followed by ligation of platform-specific adaptors at the end of each fragment. These adaptors are then used in amplification of the fragment on a solid surface by a polymerase, except in cPAL amplification, which is performed in solution. The amplification products are spatially clustered on an array before sequencing. The sequencing process itself is performed by an orchestrated automated series of enzyme-driven biochemical and fluorescent imaging data acquisition steps. Only the newest system, the Ion Torrent, based on free proton shifts, is capable of electric detection. All of these platforms also have the capability to read both ends of a DNA fragment, called paired-end sequencing. This feature is instrumental in resolving repetitive regions in genomes and quantifying transcript isoforms in RNA-Seq.

The rate-limiting step in the sequencing process has traditionally been the sequencing reaction. However, that started to change towards the end of the HGP, when the capacity of sequencing instruments began to exceed the rate at which new samples could be prepared for sequencing. Current sequencing instruments produce several orders of magnitude more data than conventional Sanger sequencing, shifting the rate-limiting steps to library preparation and data analysis. The challenge is to keep the processes of sample preparation, sequencing reaction and data analysis balanced. Hence, automation of library preparation and quality control steps has played a vital role in keeping up to speed with the increase in sequencing power. The generally short read lengths coupled to the enormous amount of data to be analyzed and reduced raw accuracy, compared to Sanger sequencing, has introduced many challenges in the downstream data analysis [18]. However, the development of new algorithms tailored to the new kinds of data generated and the use of supercomputer clusters for the data analysis have alleviated some of these challenges.

## 4 Massively parallel sequencing – single-molecule sequencing

**Figure 2:**
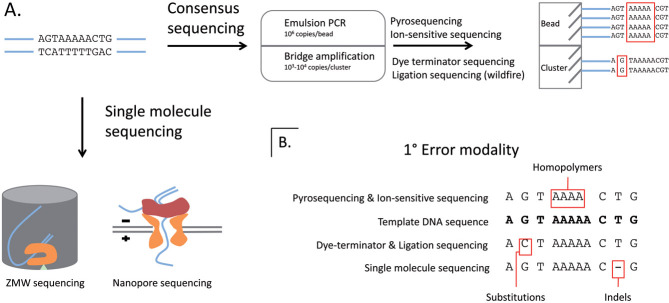
(**A**) Comparison of number of DNA molecules required for generating a base call in consensus sequencing and single-molecule sequencing. (**B**) The most common type of sequencing errors per sequencing technology.

Massively parallel consensus sequencing has become the dominant sequencing technology, but other approaches have emerged that avoid amplification of the DNA template prior to sequencing. The aim of these technologies is to sequence single DNA molecules, preferably in real time. Potential benefits of using single-molecule sequencing are: the minimal quantities of input DNA required; elimination of amplification bias; asynchronous synthesis; fast turnaround times; and the capacity to investigate the characteristics of individual DNA molecules. A comparison of consensus and single-molecule sequencing, as well as the most common errors for each sequencing technology is shown in Fig. 2.

### 4.1 Reversible single-dye terminator SBS

Helicos Biosciences introduced the first single-molecule DNA sequencer, the Heliscope sequencer system in 2008 [37]. The system uses labeled reversible chain-terminating nucleotides in an SBS approach with immobilized DNA on a planar surface [38, 39]. Hence, it shares many features with massively parallel consensus sequencers in set-up, biochemistry, sequence run time and throughput. It was used in the first single-molecule sequencing of a human genome in 2009 [40] and can be used for amplification-free direct RNA sequencing [41]. However, the platform has been on the market since 2008 and seems to be struggling in the fierce competition. The reasons for this probably lie in its the high raw read error rates (>5%) and short read length (∼32 bases) [42].

### 4.2 Zero-mode waveguide sequencing with immobilized polymerase

Pacific Biosciences presented the first single-molecule, real-time SBS sequencing platform in a proof-of-principle paper in *Science* in 2009. The platform introduced several interesting novel features, including immobilization of the DNA polymerase instead of the DNA, real-time monitoring of the synthesis process and potential read lengths of several kilobases of DNA [43].

The single-molecule real-time (SMRT) sequencing relies on a processive DNA polymerase immobilized via a biotin-streptavidin linkage at the bottom of a nanostructure called a zero-mode waveguide (ZMW) [44]. This structure is critical because it provides a confined optical observation space of ∼100 zeptoliters (100 × 10^–21^ L), enabling minimization of background noise, parallelization, and monitoring of single-molecule DNA polymerization. The DNA template is allowed to diffuse into the ZMV in the presence of primer and nucleotides with fluorescent labels attached to the phosphate chain. The DNA polymerase latches onto the DNA template and begins incorporating the distinctly labeled nucleotides. The DNA sequence is determined by recording each nucleotide incorporation event dictated by the DNA template. Nucleotides that are being incorporated remain for a longer time in the optical detection volume than freely diffusing nucleotides, facilitating the separation of background noise generated by a high concentration of labeled nucleotides from true nucleotide incorporation events. The label attached to the phosphate chain is cleaved off during the elongation process. The polymerases are randomly immobilized among the ZMWs, leading to only a third of them being occupied by a single polymerase. The rest of the ZMWs are either empty or contain two or more polymerases, which dramatically reduces the potential capacity of the ZMW array. SMRT sequencing has high error rates, mainly due to failure to detect all incorporations leading to indels [42, 43].

SMRT sequencing has several interesting features that can be exploited. A circular template can be used, enabling a high-quality consensus sequence to be obtained by allowing the polymerase to make multiple passes along the same DNA template [45]. The physical read length can be extended by turning off the laser used to illuminate the ZMWs at pre-determined intervals, generating sub-reads from the same genomic fragment in a process called strobe sequencing [46]. A truly novel feature is the ability to capture kinetic information by observing the activity of an immobilized enzyme in real time. This enables the detection of chemical modifications to bases, such as methylation [47], and the observation of translation kinetics by employing labeled tRNAs and immobilizing a ribosome instead of a polymerase in the ZMW [48]. The SMRT sequencing approach has great potential with unique features and fast sequencing reaction runs, but suffers from raw error rates exceeding 10%, largely due to indels, and modest outputs (2 × 75 000 ZMWs per SMRT cell allowing to about a third as many reads), which hamper its use in many applications.

### 4.3 Nanopore sequencing

In 1996, Deamer and colleagues demonstrated that single-stranded DNA (1.1–1.3 nm; [49]) or RNA molecules could be driven through an ion channel in a lipid bilayer by an electrical field [50]. This cited paper outlined the basic principles of a nanopore sequencing method. The DNA to be sequenced would be decoded by monitoring an electric current as the DNA passed through a nanopore (a channel 1–10 nm in diameter) in a membrane, which could be either nanofabricated or created by an engineered protein. Nanopore sequencing can potentially offer minimal sample preparation, electrical or fluorescent readout and read lengths of several kilobases from single molecules of DNA in real time [42, 51].

Several problems must be resolved before nanopore sequencing can be fully exploited. The nanopores are too long to provide single-base resolution, as several nucleotides in the pore contribute to each change in electric current. In addition, the high speed of DNA translocation through the nanopores complicates the electrical measurements, making the nucleotide identification signals difficult to interpret [42]. However, solutions to these problems have been proposed. IBM is developing a nanopore matrix, which resembles a transistor with alternating layers of metal and dielectric material. Simulations indicate that modulating the current in this “transistor” can control the speed of DNA translocation [52]. However, the effect needs to be verified experimentally and a potential device still needs to achieve single-base resolution [42, 52]. Genia is a nanopore sequencing company using a lipid bilayer with a single nanopore connected to a sensor on an integrated circuit. They aim to reach a chip containing ∼1 million such sensors for the commercial launch of their system, which is scheduled to 2013. However, there are no reports from non-vendors that have tested the Genia system yet. Another commercial player trying to achieve nanopore sequencing is Oxford Nanopore Technologies. They currently have two DNA sequencing strategies: strand and exonuclease sequencing. Exonuclease sequencing employs a modified α-hemolysin with an attached exonuclease, situated within a synthetic membrane with high electronic resistance. The exonuclease cleaves off single nucleotides and feeds them into the pores. Each individual nucleotide can then be detected from their distinct electrical signal as they transiently bind to a cyclodextrin molecule when passing through the pore [53]. Strand sequencing relies on a polymerase (or other DNA-modifying enzyme) feeding single-stranded DNA into the pore and detecting the electrical signal of three, for example, bases interacting with a specific region of the protein pore.

Recently, Oxford Nanopore Technologies announced its ability to sequence the entire 48-kilobase Lambda genome on both sense and antisense strands using their strand sequencing method. Commercialization of the nanopore-based sensing chemistry on an electronic-based platform, the GridION, and a disposable portable device for electronic single molecule sensing, the MinION, is scheduled for 2012. Oxford Nanopore Technologies claims competitive accuracy and ultra-long read-length single-molecule data, which will deliver a complete human genome in 15 min using multiple GridIONs. It is too early to tell whether Oxford Nanopore Technologies can fully deliver on these promises, and the acid test for the sequencing technique will be the assessment of the early tester. However, if the promise of competitive accuracy and ultra-long reads holds true it will cause another paradigm shift in the sequencing field.

## 5 Future perspectives

Sequencing technologies have advanced at a rapid pace in recent years and the advance shows no signs of slowing down. This has created an imbalance between the sequencing per se and other procedures involved, both upstream and downstream, i.e. sample preparation and sequence data analysis and storage. Bar coding of samples coupled to automation of steps prior to sequencing has the potential to handle most upstream process issues. The true challenges lie in the downstream steps of sequence data analysis, handling and storage. The assembly and mapping algorithms need to become more efficient and accurate to handle the massive amounts of sequence data and extract as much pertinent information from them as possible. Long-term storage of sequence data and subsequent analysis is cumbersome and expensive, thus, in the future, it might be cheaper and easier to simply re-sequence samples as the information is needed. There are, of course, several challenges remaining in the actual sequencing reactions. Two of the most urgent are increasing the read lengths and the quality of the sequence reads, enabling resolution of common repeats and duplicated regions. Currently, the massively parallel sequencing and single-molecule platforms are complementary, and no single technology is best suited for all applications. Thus, it remains to be seen which sequencing technology will cause the next sequencing paradigm shift.

Sequencing has traditionally been reserved for large and very well-funded research centers, but the cost per base sequenced started to decrease during the end of the HGP. The pace of cost reduction accelerated even further with the launch and subsequent evolution of the massively parallel sequencing instruments seen in recent years. What was once restricted to a privileged few can now be accessed by many, leading to a revolution in the availability of sequencing power.

The democratization of sequencing is evident from the constantly increasing number of applications and analyses that involve sequence data. Sequencing is today applied in diverse areas, such as forensics, agriculture, biofuel production, paleontology, domestication, and medicine to name but a few. This ongoing revolution promises to bring sequencing to nearly every aspect of life.

## Abbreviations

HGP: Human Genome Project
PPi: pyrophosphate
SBS: sequencing-by-synthesis
SMRT: single-molecule real-time
SOLiD: sequencing by oligonucleotide ligation and detection
ZMW: zero-mode waveguide

## References

[1] Maxam AM, Gilbert W (1977). A new method for sequencing DNA. Proc. Natl. Acad. Sci. USA.

[2] Sanger F, Nicklen S, Coulson AR (1977). DNA sequencing with chain-terminating inhibitors. Proc. Natl. Acad. Sci. USA.

[3] Smith LM, Sanders JZ, Kaiser RJ, Hughes P (1986). Fluorescence detection in automated DNA sequence analysis. Nature.

[4] Prober JM, Trainor GL, Dam RJ, Hobbs FW (1987). A system for rapid DNA sequencing with fluorescent chain-terminating dideoxynucleotides. Science.

[5] Cohen AS, Najarian DR, Paulus A, Guttman A (1988). Rapid separation and purification of oligonucleotides by high-performance capillary gel electrophoresis. Proc. Natl. Acad. Sci. USA.

[6] Luckey JA, Drossman H, Kostichka AJ, Mead DA (1990). High speed DNA sequencing by capillary electrophoresis. Nucleic Acids Res.

[7] Karger BL, Guttman A (2009). DNA sequencing by CE. Electrophoresis.

[8] Melamede RJ

[9] Nyren P (2007). The history of pyrosequencing. Methods Mol. Biol.

[10] Ronaghi M, Uhlen M, Nyren P (1998). A sequencing method based on real-time pyrophosphate. Science.

[11] Margulies M, Egholm M, Altman WE, Attiya S (2005). Genome sequencing in microfabricated high-density picolitre reactors. Nature.

[12] Rothberg JM, Leamon JH (2008). The development and impact of 454 sequencing. Nat. Biotechnol.

[13] Wheeler DA, Srinivasan M, Egholm M, Shen Y (2008). The complete genome of an individual by massively parallel DNA sequencing. Nature.

[14] Lander ES, Linton LM, Birren B, Nusbaum C (2001). Initial sequencing and analysis of the human genome. Nature.

[15] Green RE, Krause J, Briggs AW, Maricic T (2010). A draft sequence of the Neandertal genome. Science.

[16] Bennett ST, Barnes C, Cox A, Davies L, Brown C (2005). Toward the $1000 human genome. Pharmacogenomics.

[17] Balasubramanian S, Bentley D

[18] Shendure J, Ji H (2008). Next-generation DNA sequencing. Nat. Biotechnol.

[19] Bentley DR, Balasubramanian S, Swerdlow HP, Smith GP (2008). Accurate whole human genome sequencing using reversible terminator chemistry. Nature.

[20] Bentley DR (2006). Whole-genome re-sequencing. Curr. Opin. Genet. Dev.

[21] Sanger F, Coulson AR (1975). A rapid method for determining sequences in DNA by primed synthesis with DNA polymerase. J. Mol. Biol.

[22] Li R, Zhu H, Ruan J, Qian W (2010). De novo assembly of human genomes with massively parallel short read sequencing. Genome Res.

[23] Li R, Fan W, Tian G, Zhu H (2010). The sequence and de novo assembly of the giant panda genome. Nature.

[24] Lehnman IR (1974). DNA Ligase: Structure, mechanism, and function. Science.

[25] Shendure J, Porreca GJ, Reppas NB, Lin X (2005). Accurate multiplex polony sequencing of an evolved bacterial genome. Science.

[26] Metzker ML (2010). Sequencing technologies – the next generation. Nat. Rev. Genet.

[27] McKernan KJ, Peckham HE, Costa GL, McLaughlin SF (2009). Sequence and structural variation in a human genome uncovered by short-read, massively parallel ligation sequencing using two-base encoding. Genome Res.

[28] Valouev A, Ichikawa J, Tonthat T, Stuart J (2008). A high-resolution, nucleosome position map of *C. elegans* reveals a lack of universal sequence-dictated positioning. Genome Res.

[29] Bao S, Jiang R, Kwan W, Wang B (2011). Evaluation of next-generation sequencing software in mapping and assembly. J. Hum. Genet.

[30] Drmanac R, Sparks AB, Callow MJ, Halpern AL (2010). Human genome sequencing using unchained base reads on self-assembling DNA nanoarrays. Science.

[31] Fire A, Xu SQ (1995). Rolling replication of short DNA circles. Proc. Natl. Acad. Sci. USA.

[32] Pourmand N, Karhanek M, Persson HHJ, Webb CD (2006). Direct electrical detection of DNA synthesis. Proc. Natl. Acad. Sci. USA.

[33] Rothberg JM, Hinz W, Rearick TMD, Schultz J An integrated semiconductor device enabling non-optical genome sequencing. Nature.

[34] Toumazou C, Premanode B, Shepherd L

[35] Mellmann A, Harmsen D, Cummings CA, Zentz EB (2011). Prospective genomic characterization of the German enterohemorrhagic *Escherichia coli* O104:H4 outbreak by rapid next generation sequencing technology. PLoS ONE.

[36] Rohde H, Qin J, Cui Y, Li D (2011). Open-source genomic analysis of shiga-toxin-producing *E. coli* O104:H4. N. Engl. J. Med.

[37] Efcavitch JW, Thompson JF (2010). Single-molecule DNA analysis. Annu. Rev. Anal. Chem. (Palo Alto Calif).

[38] Harris TD, Buzby PR, Babcock H, Beer E (2008). Single-molecule DNA sequencing of a viral genome. Science.

[39] Bowers J, Mitchell J, Beer E, Buzby PR (2009). Virtual terminator nucleotides for next-generation DNA sequencing. Nat. Methods.

[40] Pushkarev D, Neff NF, Quake SR (2009). Single-molecule sequencing of an individual human genome. Nat. Biotechnol.

[41] Ozsolak F, Platt AR, Jones DR, Reifenberger JG (2009). Direct RNA sequencing. Nature.

[42] Schadt EE, Turner S, Kasarskis A (2010). A window into third-generation sequencing. Hum. Mol. Genet.

[43] Eid J, Fehr A, Gray J, Luong K (2009). Real-time DNA sequencing from single polymerase molecules. Science.

[44] Korlach J, Bjornson KP, Chaudhuri BP, Cicero RL (2010). Real-time DNA sequencing from single polymerase molecules. Methods Enzymol.

[45] Travers KJ, Chin C-S, Rank DR, Eid JS, Turner SW (2010). A flexible and efficient template format for circular consensus sequencing and SNP detection. Nucleic Acids Res.

[46] Lo C, Bashir A, Bansal V, Bafna V (2011). Strobe sequence design for haplotype assembly. BMC Bioinformatics.

[47] Flusberg BA, Webster DR, Lee JH, Travers KJ (2010). Direct detection of DNA methylation during single-molecule, real-time sequencing. Nat. Methods.

[48] Uemura S, Aitken CE, Korlach J, Flusberg BA (2010). Real-time tRNA transit on single translating ribosomes at codon resolution. Nature.

[49] Mandelkern M, Elias JG, Eden D, Crothers DM (1981). The dimensions of DNA in solution. J. Mol. Biol.

[50] Kasianowicz JJ, Brandin E, Branton D, Deamer DW (1996). Characterization of individual polynucleotide molecules using a membrane channel. Proc. Natl. Acad. Sci. USA.

[51] Branton D, Deamer DW, Marziali A, Bayley H (2008). The potential and challenges of nanopore sequencing. Nat. Biotechnol.

[52] Luan B, Peng H, Polonsky S, Rossnagel S (2010). Base-by-base ratcheting of single stranded DNA through a solid-state nanopore. Phys. Rev. Lett.

[53] Clarke J, Wu H-C, Jayasinghe L, Patel A (2009). Continuous base identification for single-molecule nanopore DNA sequencing. Nat. Nanotechnol.

